# Endocarditis-related tibioperoneal trunk pseudoaneurysm managed with endovascular treatment - case report

**DOI:** 10.1590/1677-5449.010217

**Published:** 2018

**Authors:** Marcio Miyamotto, Gabriele Simões Marcusso, Thalita Toldo Ferreira, Matheus Schimidt Evangelista, Giana Caroline Strack Neves, Ian Gimenez Ribeiro, Cintia Lopes Raymundo, Fernanda Reis Gomes

**Affiliations:** 1 Hospital Universitário Cajuru – HUC, Pontifícia Universidade Católica do Paraná – PUC-PR, Serviço de Cirurgia Vascular e Endovascular, Curitiba, PR, Brasil.; 2 Instituto VESSEL de Aperfeiçoamento Endovascular de Curitiba, Curitiba, PR, Brasil.; 3 Hospital Nossa Senhora das Graças - HNSG, Serviço de Cirurgia Vascular e Endovascular Elias Abrão, Curitiba, PR, Brasil.; 4 Liga Acadêmica de Medicina Vascular - LAMEV, Hospital Universitário Cajuru – HUC, Pontifícia Universidade Católica do Paraná – PUC-PR, Curitiba, PR, Brasil.; 5 Hospital Santa Cruz, Serviço de Cirurgia Vascular, Curitiba, PR, Brasil.

**Keywords:** pseudoaneurysm, tibioperoneal trunk, endocarditis, endovascular

## Abstract

Tibioperoneal trunk aneurysms are rare and the majority of them are pseudoaneurysms This report describes an unusual case of a pseudoaneurysm secondary to bacterial endocarditis diagnosed and treated several years previously. After ruling out ongoing infection, the patient was successfully treated by percutaneous covered stent implantation. In this scenario, the use of endovascular techniques offered a safe and effective alternative treatment.

## INTRODUCTION

 The term mycotic aneurysm was used for the first time in 1885 by Sir William Osler in his work on bacterial endocarditis. At that time, distal septic embolization was found in up to 80% of cases and was considered to be related to pseudoaneurysm formation. 1 This was confirmed by the sudden reduction in the incidence of this complication after the introduction of antibiotic therapy and techniques for replacement of infected heart valves to treat endocarditis. 2

 The majority of cases of endocarditis-related distal arterial embolization involve the bifurcation of the common femoral artery. 2 Few cases involving infragenicular arteries have been described. 2 ^-^ 7 This article describes the case of a pseudoaneurysm of the tibioperoneal trunk with late presentation after treatment for bacterial endocarditis. In this case, after ruling out any type of active infectious process, we decided to manage this patient with a covered stent implantation. 

## CASE DESCRIPTION

 The patient was a 65-year-old male who presented with a swelling of the posterior surface of the proximal third of his left leg. His prior history included a prolonged stay in hospital for treatment of bacterial endocarditis, when two mitral valve replacement operations were performed. He had also previously undergone two abdominal operations to treat an intestinal tumor and one varicose veins surgery. 

 Physical examination revealed a pulsating mass in the posterior region of the proximal third of the left leg. Femoral, popliteal, and dorsal pedal pulses were palpable and normal in both lower limbs. The posterior tibial artery pulse was absent, whereas the posterior tibial artery pulse was palpable in the right lower limb. 

**Figure 1:**
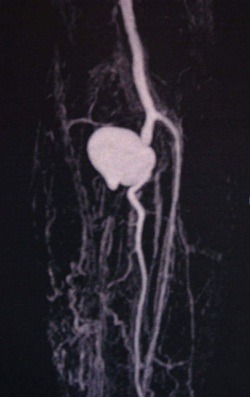
Magnetic resonance angiography of the left lower limb showing pseudoaneurysm of the tibioperoneal trunk and occlusion of the posterior tibial artery.

 Magnetic resonance angiography showed a saccular dilatation in the tibioperoneal trunk with a 4.4 cm diameter, at the level of the origin of the posterior tibial artery. The posterior tibial artery was also occluded ( Figure 1 ). Investigation was supplemented with laboratory tests (inflammatory activity tests, coagulation tests, and complete blood cell count), which all returned normal results, in addition to blood cultures, which were negative. 

**Figure 2:**
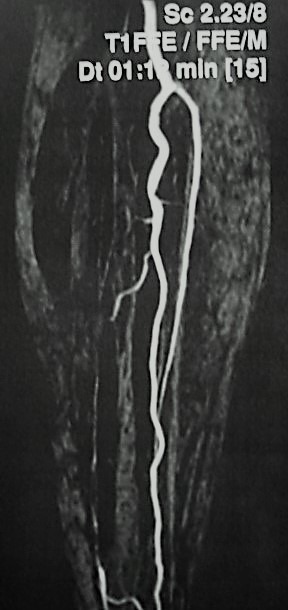
Control magnetic resonance angiography showing no perfusion of the aneurysm sac and a patent fibular artery.

 Having ruled out other probable etiologies and active infections, in view of the history of bacterial endocarditis, it was decided to perform a less invasive treatment, considering the inflammatory/infectious pathophysiology and the size and site of the pseudoaneurysm. A covered, self-expanding stent (Gore VIABAHN 6.0 × 50 mm) was therefore placed in the tibioperoneal trunk, preserving fibular artery patency and excluding the aneurysm. The transition between the tibioperoneal trunk and the fibular artery was ectatic, because of the pseudoaneurysm, which minimized difficulties caused by reduction of the distal diameter. A control magnetic resonance angiography showed that the aneurysm sac was no longer perfused and the fibular artery was patent ( Figure 2 ). 

 The patient was followed up for 10 years after treatment and during this period the symptoms did not recur and the patient maintained an ankle-brachial index of 1.0. 

## DISCUSSION

 Infrapopliteal peripheral aneurysms are rare and, when they do occur, they are generally pseudoaneurysms. There are few cases described in the literature, most of which are related to arterial traumatisms (such as fractures of the tibia or malleolus), peripheral venous access, percutaneous transluminal angioplasty, or embolization secondary to bacterial endocarditis. 3 This last group is even rarer because of use of antibiotics and early surgical treatment for bacterial endocarditis and, when they do occur, they tend to affect axial arteries such as the aorta, cerebral arteries, mesenteric arteries, and the common femoral artery more often. 4

 Mycotic pseudoaneurysms can develop after infection of the vessel or they may be caused by a secondary infection of a preexisting aneurysm. Both are rare and their incidence ranges from 1 to 3.7%, considering saccular aneurysms. 7 Despite what the name suggests, mycotic aneurysms are generally secondary to bacterial infections, most often by *Staphylococcus aureus* or *Salmonella spp* . They can also be caused by fungal infections, but this is very rare, occurring in 1 to 2% of cases. 3 ^,^ 7

 The physiopathogenesis of mycotic pseudoaneurysms is related to septic microembolizations involving the *vasa vasorum*, leading to ischemia, focal necrosis and weakness of the artery wall, and resulting in rupture and formation of the pseudoaneurysm. In the majority of reports in the literature, these embolizations generate pseudoaneurysms in axial vessels such as the aorta, mesenteric arteries, cerebral vessels, and the femoral arteries, rarely involving infrapopliteal territories, to the extent that just 10 cases are described in literature published up to 2012. 2 ^,^ 4 ^-^ 7

 Clinical presentation is variable and patients may be asymptomatic or may have symptoms including edema and pain and a sensation of a pulsating mass in the region involved. Symptoms related to nerve and vein compression depend on the size and site of the pseudoaneurysm. Rupture and distal embolization are extremely dangerous and urgent situations and can be exacerbated further still in the presence of active infection. 8

 Early recognition and treatment of a mycotic pseudoaneurysm are essential for preventing complications and management should be decided on a case-by-case basis. Traditionally, treatment consists of resection of the pseudoaneurysm with debridement of necrotic and infected tissues, ligature of the affected vessel and revascularization of the limb, which should preferably be performed with an autologous graft, via an anatomic or extra-anatomic route, and followed by a prolonged period of broad spectrum antibiotic therapy. 6 In 1992, Donald et al. reported on a case of a mycotic pseudoaneurysm of the tibioperoneal trunk with onset 18 months after bacterial endocarditis caused by *Streptococcus viridans* that was successfully managed by open repair, with restoration of the distal arterial circulation. 6 Since then, other cases of pseudoaneurysms in this topography have been reported in the literature and management with traditional surgical techniques remained the first choice. 2 ^-^ 7

 Endovascular techniques were first used to repair peripheral pseudoaneurysms in 1994. Geremia et al. placed endovascular stents in pseudoaneurysms that had been induced in the carotid arteries of dogs, observing thrombosis of the pseudoaneurysm sac and patency of the vessel involved. 9

 However, there are few reports in the literature on use of endovascular techniques to treat infrapopliteal pseudoaneurysms. Sadat et al. used coil embolization in a case of pseudoaneurysm of the fibular artery after embolectomy with a fogarty catheter. 8 They achieved total occlusion of flow into the pseudoaneurysm and the patient was discharged from hospital free from symptoms, 2 days after the procedure. 8 Larena-Avellaneda et al. also conducted coil embolization to treat a patient with mycotic pseudoaneurysm after endocarditis caused by *Candida albicans*. 7 In the case reported here, an endovascular technique was employed to place a flexible covered stent and exclude the pseudoaneurysm. No similar cases were found in the literature. The late onset of the pseudoaneurysm contributed to making use of this technique possible, since there was no active infection, enabling implantation of a prosthetic material. 

## CONCLUSIONS

 Pseudoaneurysms of infectious etiology are rare. In the majority of cases, the treatment of choice is resection combined with revascularization of the territory supplied by the vessel involved. Late-onset mycotic pseudoaneurysms without active infections can be managed using endovascular techniques, particularly in cases with atypical locations or where surgical access is more restricted. 
