# Supplementary material for: Endocarditis-related tibioperoneal trunk pseudoaneurysm managed with endovascular treatment - case report
Source: J Vasc Bras. 2018 Oct-Dec;17(4):318–21. doi: 10.1590/1677-5449.010217 (PMC6375271; doi:10.1590/1677-5449.010217)
Supplement: Translation

# Translation: Correção endovascular de pseudoaneurisma de tronco tibiofibular secundário a endocardite – relato de caso

**Authors:** Marcio Miyamotto, Gabriele Simões Marcusso, Thalita Toldo Ferreira, Matheus Schimidt Evangelista, Giana Caroline Strack Neves, Ian Gimenez Ribeiro, Cintia Lopes Raymundo, Fernanda Reis Gomes

**Affiliations:** 1 Hospital Universitário Cajuru – HUC, Pontifícia Universidade Católica do Paraná – PUC-PR, Serviço de Cirurgia Vascular e Endovascular, Curitiba, PR, Brasil.; 2 Instituto VESSEL de Aperfeiçoamento Endovascular de Curitiba, Curitiba, PR, Brasil.; 3 Hospital Nossa Senhora das Graças - HNSG, Serviço de Cirurgia Vascular e Endovascular Elias Abrão, Curitiba, PR, Brasil.; 4 Liga Acadêmica de Medicina Vascular - LAMEV, Hospital Universitário Cajuru – HUC, Pontifícia Universidade Católica do Paraná – PUC-PR, Curitiba, PR, Brasil.; 5 Hospital Santa Cruz, Serviço de Cirurgia Vascular, Curitiba, PR, Brasil.

**Keywords:** pseudoaneurisma, tronco tibiofibular, endocardite, endovascular

## Abstract

Os aneurismas do tronco tibiofibular são raros e consistem principalmente em pseudoaneurismas. Os autores descrevem um caso incomum de pseudoaneurisma do tronco tibiofibular secundário a endocardite bacteriana diagnosticada e tratada vários anos antes. Após a exclusão de um processo infeccioso ativo, o paciente foi tratado com sucesso através do implante percutâneo de um stent recoberto. O tratamento endovascular, neste contexto, apresentou uma alternativa segura e eficaz.

## INTRODUÇÃO

 O termo aneurisma micótico foi utilizado pela primeira vez por Sir William Osler, em 1885, em sua dissertação sobre endocardite bacteriana. Naquela ocasião, foi identificada a ocorrência de embolização séptica distal em até 80% dos casos, a qual seria responsável pela formação desses pseudoaneurismas 1 . Esse fato pode ser comprovado pela súbita diminuição da incidência dessa complicação após o advento da antibioticoterapia e das técnicas de substituição das válvulas cardíacas infectadas no tratamento das endocardites 2 . 

 A maioria dos casos de embolização arterial distal relacionada à endocardite ocorre na bifurcação da artéria femoral comum 2 . Poucos casos foram descritos envolvendo as artérias infrageniculares 2 ^-^ 7 . No presente artigo, relatamos um caso de pseudoaneurisma de tronco tibiofibular com apresentação tardia ao tratamento de endocardite bacteriana. Nesse caso, após exclusão de qualquer processo infeccioso ativo, optamos pelo implante de um stent recoberto como forma de tratamento. 

## DESCRIÇÃO DO CASO

 Paciente do sexo masculino, 65 anos, apresentou queixa de aumento de volume na face posterior do terço proximal da perna esquerda. Possuía história pregressa de internamento prolongado para tratamento de endocardite bacteriana, sendo realizadas, nessa ocasião, duas cirurgias para troca valvar mitral. Apresentava também duas cirurgias abdominais prévias por tumor intestinal e uma cirurgia de varizes. 

 Ao exame físico, apresentava uma massa pulsátil na região posterior do terço proximal da perna esquerda. Os pulsos femoral, poplíteo e dorsal do pé de ambos os membros inferiores eram palpáveis e simétricos. O pulso da artéria tibial posterior do membro inferior esquerdo era impalpável, enquanto o pulso da artéria tibial posterior do membro inferior direito era palpável. 

 Foi solicitada uma angiorressonância, que evidenciou a presença de uma dilatação de formato sacular, medindo 4,4 cm em seu maior diâmetro, no tronco tibiofibular do membro inferior esquerdo, ao nível da origem da artéria tibial posterior. A artéria tibial posterior encontrava-se ocluída ( Figura 1 ). Para investigação complementar, foram solicitados exames laboratoriais (provas de atividade inflamatória, coagulograma e hemograma completo), que apresentaram resultados normais, além de hemoculturas, que foram negativas. 

**Figura 1:** Angiorressonância de membro inferior esquerdo evidenciando pseudoaneurisma em tronco tibiofibular e oclusão da artéria tibial posterior.

 Afastadas outras prováveis etiologias ou infecção ativa devido ao histórico de endocardite bacteriana, optou-se por um tratamento menos invasivo considerando a fisiopatologia inflamatória/infecciosa do processo, o tamanho e a localização do pseudoaneurisma. O paciente foi então submetido ao implante de um stent recoberto autoexpansível (Gore VIABAHN 6.0 × 50 mm) no tronco tibiofibular, mantendo a perviedade da artéria fibular e excluindo o aneurisma. Devido à presença do pseudoaneurisma, a transição entre o tronco tibiofibular e a artéria fibular encontrava-se ectasiada, minimizando a dificuldade relacionada à redução de diâmetro distal. A angiorressonância de controle evidenciou a ausência de perfusão do saco aneurismático, além da perviedade da artéria fibular ( Figura 2 ). 

**Figura 2:** Angiorressonância de controle evidenciando ausência de perfusão do saco aneurismático e perviedade da artéria fibular.

 O paciente foi seguido por um período de 10 anos após o tratamento, não apresentando recorrência dos sintomas durante o período e mantendo índice tornozelo-braquial (ITB) de 1,0. 

## DISCUSSÃO

 Os aneurismas periféricos de localização infrapoplítea são raros e, quando ocorrem, geralmente são pseudoaneurismas. Existem poucos casos descritos na literatura, sendo comumente relacionados a traumatismos arteriais (como fraturas tibiais ou maleolares), acessos venosos periféricos, angioplastia transluminal percutânea ou embolizações secundárias a endocardite bacteriana 3 . Estes são ainda mais raros devido ao uso de antibióticos e procedimentos cirúrgicos precoces no tratamento da endocardite bacteriana e, quando ocorrem, costumam acometer mais comumente artérias axiais como a aorta, artérias cerebrais, artérias mesentéricas e a artéria femoral comum 4 . 

 Os pseudoaneurismas micóticos podem se desenvolver após a infecção do vaso ou podem ser resultado da infecção secundária de um aneurisma pré-existente. Ambos são raros e apresentam uma incidência entre 1 e 3,7%, considerando os aneurismas de configuração sacular 7 . Ao contrário do que o nome sugere, os aneurismas micóticos geralmente são secundários a uma infecção bacteriana, mais comumente por *Staphylococcus aureus* e *Salmonella spp*. Também podem ocorrer como consequência de infecção por fungos, embora seja muito incomum, ocorrendo em 1 a 2% dos casos 3 ^,^ 7 . 

 A fisiopatogenia do pseudoaneurisma micótico está relacionada a microembolizações sépticas que acometem os *vasa vasorum*, levando a isquemia, necrose focal e fragilidade da parede arterial, resultando em ruptura e formação do pseudoaneurisma. Na maioria dos relatos na literatura, tais embolizações geram pseudoaneurismas em vasos axiais como aorta, artérias mesentéricas, vasos cerebrais e artéria femoral, raramente atingindo o território infrapoplíteo, sendo que existiam apenas 10 casos descritos na literatura até 2012 2 ^,^ 4 ^-^ 7 . 

 A apresentação clínica é variável, podendo o paciente ser assintomático ou apresentar queixas de edema, dor e sensação de massa pulsátil na região acometida. Os sintomas relacionados à compressão nervosa e venosa são dependentes do tamanho e da localização do pseudoaneurisma. A ruptura e a embolização distal são complicações de extrema urgência e gravidade, agravadas ainda mais quando há presença de processo infeccioso ativo 8 . 

 O reconhecimento e o tratamento precoce do pseudoaneurisma micótico são essenciais para a prevenção de complicações, cujo manejo deve ser individualizado a cada caso. Tradicionalmente, o tratamento consiste na ressecção do pseudoaneurisma com debridamento dos tecidos necróticos e infectados, ligadura do vaso acometido e revascularização do membro, que deve ser realizada preferencialmente com enxerto autógeno, por via anatômica ou extra-anatômica, seguida de um período prolongado de antibioticoterapia 6 . Em 1992, Donald et al. relataram um caso de pseudoaneurisma micótico em tronco tibiofibular que surgiu 18 meses após um quadro de endocardite bacteriana por *Streptococcus viridans* e foi manejado com sucesso por reparo aberto, com restituição da circulação arterial distal 6 . Após isso, outros casos de pseudoaneurisma nessa topografia também foram relatados na literatura, mantendo o manejo com a técnica cirúrgica tradicional como primeira escolha 2 ^-^ 7 . 

 O uso de técnicas endovasculares para tratamento de pseudoaneurismas periféricos se iniciou em 1994. Geremia et al. implantaram stents endovasculares em pseudoaneurismas induzidos em artérias carótidas de cães e observaram trombose do saco do pseudoaneurisma e perviedade do vaso acometido 9 . 

 Entretanto, o uso da técnica endovascular para o tratamento de pseudoaneurismas infrapoplíteos possui poucos relatos na literatura. Sadat et al. realizaram embolização com molas em um caso de pseudoaneurisma de artéria fibular pós-embolectomia com cateter de Fogarty 8 . Houve oclusão total do fluxo no pseudoaneurisma, e o paciente recebeu alta hospitalar assintomático, dois dias após o procedimento 8 . Larena-Avellaneda et al. também realizaram a embolização com molas para o tratamento de um paciente com pseudoaneurisma micótico após endocardite por *Candida albicans* 7 . No caso aqui relatado, foi utilizada a técnica endovascular com colocação de stent revestido flexível para exclusão do pseudoaneurisma, sendo que não foram encontrados casos semelhantes na literatura. A apresentação tardia do pseudoaneurisma contribuiu para que fosse possível a utilização dessa técnica, já que não havia infecção ativa na ocasião, permitindo, assim, o implante de um material protético. 

## CONCLUSÃO

 Os pseudoaneurismas de etiologia infecciosa são raros. O tratamento de escolha, na maioria dos casos, é a ressecção associada à revascularização do território perfundido por esse vaso. Pseudoaneurismas micóticos de apresentação tardia sem infecção ativa podem ser manejados com técnicas endovasculares, principalmente nos casos de localizações atípicas ou de acesso cirúrgico mais restrito.
